# Cathelicidins from the Bullfrog *Rana catesbeiana* Provides Novel Template for Peptide Antibiotic Design

**DOI:** 10.1371/journal.pone.0093216

**Published:** 2014-03-27

**Authors:** Guiying Ling, Jiuxiang Gao, Shumin Zhang, Zeping Xie, Lin Wei, Haining Yu, Yipeng Wang

**Affiliations:** 1 College of Pharmaceutical Sciences, Soochow University, Suzhou, Jiangsu, China; 2 Department of Bioscience and Biotechnology, Dalian University of Technology, Dalian, China; 3 Binzhou Medical University of Pharmaceutical College, Yantai, Shandong, China; 4 Department of Biology, Guizhou Normal University, Guiyang, Guizhou, China; National Research Council of Italy, Italy

## Abstract

Cathelicidins, a class of gene-encoded effector molecules of vertebrate innate immunity, provide a first line of defense against microbial invasions. Although cathelicidins from mammals, birds, reptiles and fishes have been extensively studied, little is known about cathelicidins from amphibians. Here we report the identification and characterization of two cathelicidins (cathelicidin-RC1 and cathelicidin-RC2) from the bullfrog *Rana catesbeiana*. The cDNA sequences (677 and 700 bp, respectively) encoding the two peptides were successfully cloned from the constructed lung cDNA library of *R. catesbeiana*. And the deduced mature peptides are composed of 28 and 33 residues, respectively. Structural analysis indicated that cathelicidin-RC1 mainly assumes an amphipathic alpha-helical conformation, while cathelicidin-RC2 could not form stable amphipathic structure. Antimicrobial and bacterial killing kinetic analysis indicated that the synthetic cathelicidin-RC1 possesses potent, broad-spectrum and rapid antimicrobial potency, while cathelicidin-RC2 exhibited very weak antimicrobial activity. Besides, the antimicrobial activity of cathelicidin-RC1 is salt-independent and highly stable. Scanning electron microscopy (SEM) analysis indicated that cathelicidin-RC1 kills microorganisms through the disruption of microbial membrane. Moreover, cathelicidin-RC1 exhibited low cytotoxic activity against mammalian normal or tumor cell lines, and low hemolytic activity against human erythrocytes. The potent, broad-spectrum and rapid antimicrobial activity combined with the salt-independence, high stability, low cytotoxic and hemolytic activities make cathelicidin-RC1 an ideal template for the development of novel peptide antibiotics.

## Introduction

Living in an environment surrounded by diverse microbial pathogens, multicellular organisms are permanently under threats [1]. Their successful survival depends on prompt and effective immune response to microbial invasions. Innate immune system forms the first line of defensive barrier against microbial invasion and proliferation [2]. One key component for innate immune system to mitigate microbial attack is antimicrobial peptides (AMPs). AMPs are a group of gene-encoded, small, and cationic peptides that possess microbicidal activities against microorganisms [3]. They are evolutionarily ancient weapons and widely distributed throughout the life kingdom [1], [4]. AMPs typically are rich in basic amino acids and adopt amphipathic conformations. They usually have a broad spectrum activity against bacteria, fungi, enveloped viruses, and even parasites [5]. Unlike most traditional antibiotics, AMPs target to the microbial membrane by electrostatic adsorption and subsequently result in cell rupture. This process occurs so quickly that it can be completed in several minutes, as a result it is unlikely for microbes to evolve resistance. Due to the high efficiency and low possibility to induce resistance, AMPs have attracted great attentions as a new generation of antibiotics.

Cathelicidins are a family of multifunctional AMPs found exclusively in vertebrates so far, including mammals [6], birds [7]–[9], reptiles [10], [11], amphibians [12]–[14] and fishes [15], [16]. Since the first discovery of Bac5 from bovine neutrophils [17], a large number of cathelicidins have been identified. In general, cathelicidins are synthesized as precursors, which possess a conserved structural organization, including a N-terminal signal peptide (30 residues), a highly conserved cathelin domain (99–114 residues) and a extremely heterogenic C-terminal mature peptide (12–100 residues) [18]. Upon stimulation, the precursors are proteolytically processed to release the cathelin domains and mature peptides, and the mature peptides then exert their antimicrobial and immunomodulatory effects [19]. Most of cathelicidins studied so far exhibit potent antimicrobial activities against a wide range of microorganisms, including a large number of clinically isolated drug-resistant pathogens. Therefore, they are regarded as ideal templates for the design of novel antibiotics.

To date, only four amphibian cathelicidins have been identified, they are cathelicidin-AL from *Amolops loloensis*, cathelicidin-PY from *Paa yunnanensis* and Lf-CATH1 and 2 from *Limnonectes fragilis* [12]–[14]. In the present study, the identification and characterization of another two novel amphibian cathelicidins, named cathelicidin-RC1 and cathelicidin-RC2 from the bullfrog *Rana catesbeiana*, was reported. The two cathelicidins were chemically synthesized, their structures, functions and antimicrobial mechanisms were extensively studied.

## Materials and Methods

### Frog collection and tissue preparation

An adult specimen of *R. catesbeiana* (weight = 260 g) was captured from Yantai, China (37.428°N 121.415°E). No specific permissions were required for the sampling location/activity, and the present study did not involve endangered or protected species. After collection, the frog was killed with a needle and the lung was quickly removed, stored in liquid nitrogen until use. The animal experimental protocols were approved by the Animal Care and Use Ethics Committee of Soochow University.

### cDNA library construction and screening of cDNAs encoding cathelicidins

The lung of *R. catesbeiana* stored in liquid nitrogen was grinded into powder and the total RNA was extracted using Trizol reagent (Life Technologies, CA, USA). The RNA was then used for cDNA library construction by an In-Fusion SMARTer^™^ Directional cDNA Library Construction Kit (Clotech, Palo Alto, CA, USA). First-strand cDNA synthesis was carried out using SMARTScribe^™^ Reverse Transcriptase (Clotech) and SMARTer V Oligonucleotide and 3′ IF SMARTer CDS Primer. Second-strand cDNA synthesis was performed by a long-distance PCR method using Advantage 2 Polymerase Mix (Clontech) in the presence of 5′ PCR Primer II A and 3′ IF SMARTer PCR Primer. The synthesized second-strand cDNAs was used as template for the following PCR-based cDNAs screening.

According to the conserved nucleotide sequence of cathelin domain of previously characterized cathelicidins, an antisense degenerate primer (5'-WSCRCAGRYCTTCACCTCC-3') was designed and coupled with a 5′ sense primer (5'-AAGCAGTGGTATCAACGCAGAGT-3') designed according to the sequence of SMARTer V Oligonucleotide to screen the 5′ fragments of cDNAs encoding cathelicidins. The PCR procedure was: 5 min of denaturation at 94°C; 30 cycles: denaturation at 94°C for 30 s, primer annealing at 57°C for 30 s, extension at 72°C for 1 min. The last cycle was followed by an extension step at 72°C for 10 min. The PCR product was purified by gel electrophoresis, cloned into pMD19-T vector (Takara, Japan) for sequencing.

After the 5′ fragments of cDNAs had been obtained, a sense primer (5'-GGATGAAGATCTGGCAGTGTGTG-3') was designed based on the 5′-coding region of cDNAs and coupled with a 3′ antisense primer (5′-TACGCGACGCGATACGCGAAT-3′) designed according to the sequence of 3′ IF SMARTer CDS Primer to screen the full length cDNAs encoding cathelicidins. The PCR procedure was: 5 min of denaturation at 94°C; 30 cycles: denaturation at 94°C for 30 s, primer annealing at 56°C for 30 s, extension at 72°C for 1 min. The last cycle was followed by an extension step at 72°C for 10 min and the PCR product was finally sequenced.

### Multi-sequence alignment and phylogenetic tree construction

Cathelicidin sequences were obtained from the protein database at the National Center for Biotechnology Information. Multi-sequence alignment was performed using Vector NTI Suite 9 program. The phylogenetic tree was constructed by the Neighbor-joining method, using MEGA program (version 5.0; www.megasoftware.net). All the cathelicidin sequences used for the phylogenetic tree construction only contain signal peptide and cathelin domain, because the mature peptides are divergent and unsuitable for evolutionary analysis. A total of 1000 bootstrap replicates were used to test the reliability of each branch. The numbers on the branches indicate the percentage of 1000 bootstrap samples supporting the branch.

### Bioinformatic analysis and structure modeling

Physical and chemical parameters of the deduced cathelicidin-RCs were analyzed through ExPASy Bioinformatics Resource Portal (http://www.expasy.org/tools/).

Homology structure modeling of cathelicidin-RCs was conducted according to the previous paper [20]. The BLAST against PDB protein databank for cathelicidin-RC1 and cathelicidin-RC2 was performed to obtain the most suitable templates. Based on the maximum sequence similarity, the crystal structures of *E. coli* ribonucleotide reductase (PDB entry 2AV8) and human sorbitol dehydrogenase (PDB entry 1PL6) deposited in the Protein Data Bank (PDB) was used as templates for homology modeling of cathelicidin-RC1 and cathelicidin-RC2, respectively. The comparative 3-D structure models of cathelicidin-RC1 and cathelicidin-RC2 were generated and optimized by homology modeling program MODELLER. The generated 3-D structural models were visualized by Pymol software (http://www.pymol.org) without any refinement.

### Peptide synthesis

Cathelicidin-RC1 and cathelicidin-RC2 were synthesized by solid phase synthesis on an Applied Biosystems model 433A peptide synthesizer following the manufacturer's standard protocols. After cleavage and deprotection of side-chain, the crude peptides were further subject to oxidation to form an intrapeptide disulphide bridge and then purified on a Vydac C18 RP-HPLC column (25 cm×1 cm). Elution was carried out at a flow rate of 1 ml/min by a linear gradient of acetonitrile in 0.1% trifluoroacetic acid in water. Identity of the synthetic peptides was confirmed by automated Edman degradation method and MALDI-TOF-MS analysis. Purity of the synthetic peptides was confirmed to be higher than 98%.

### Circular dichroism spectroscopy

Circular dichroism spectroscopy was performed using a Jasco J-715 spectrophotometer (Jasco, Japan) to evaluate the secondary structure of cathelicidin-RC1 in solvent environment. The experiment was carried out according to the method described in our previous paper with minor modifications [10]. Briefly, samples were prepared by dissolving the peptide powder to a concentration of 0.5 mg/ml in sodium dodecyl sulfate (SDS)/H_2_O solutions of different concentrations (0, 30, 60, 90, 120 mM). The spectra were measured at 298 K between 190 and 250 nm using 0.1 cm path-length cell with 1 nm bandwidth, 1 sec response time, and a scan speed of 100 nm/min. Three consecutive scans per sample were performed and averaged, followed by subtraction of the solvent signal.

### Antimicrobial assay

A two-fold broth microdilution method was used to determine the antimicrobial activity of cathelicidin-RCs as described in our previous paper [21], [22]. Briefly, microbes were incubated in Mueller-Hinton broth (MH broth) at 37°C to exponential phase and diluted with fresh MH broth to 10^6^ CFU/ml. Aliquots (50 μl) of serial dilutions of cathelicidin-RCs in MH broth were prepared in 96-well microtiter plates and mixed with equal volume of microbe inoculum. The plates were slowly shaken at 37°C for 18 h and the minimal concentrations at which no visible growth of microbes occurred were recorded as MIC values. Two traditional antibiotics (ampicillin, meropenem) were used as positive control.

### Bacterial killing kinetic assay

The bacterial killing kinetics of cathelicidin-RC1 against *E. coli* ATCC25922 was determined by measuring the changes in the viable bacterial counts after peptide treatment. The experiment was carried out according to the method described previously with minor modifications [10]. *E. coli* ATCC25922 was incubated in MH broth at 37°C for 12 h and diluted to 10^6^ CFU/ml in fresh MH broth. Cathelicidin-RC1 was added to the bacterial suspension to a final concentration of 5×MIC, and the bacteria was incubated at 37°C for 0, 10, 20, 30, 45, 60, 90, 120, and 180 mins. At each time point, aliquots (50 μl) were removed and diluted with fresh MH broth for 1000 times. 50 μl of the dilutions were coated on MH agar plates, incubated overnight at 37°C, and the viable colonies were counted. Meropenem was used as positive control and sterile deionized water was used as negative control.

### Salt tolerance, thermal tolerance and thermal stability

Salt tolerance of cathelicidin-RC1 was examined as previously described with minor modifications [23]. *E. coli* ATCC25922 was incubated in MH broth at 37°C for 12 h and diluted to 10^6^ CFU/ml in fresh MH broth, supplemented with sodium chloride at final concentrations of 0, 50, 100, 150, 200 and 400 mM, respectively. Serial dilutions of cathelicidin-RC1 dissolved in corresponding MH broth of different sodium chloride concentration were added to the *E. coli* suspension and the bacteria were incubated at 37°C for 18 h before the MICs were recorded.

Thermal tolerance of cathelicidin-RC1 was determined by measuring MICs of peptides after incubation at different temperatures. Briefly, cathelicidin-RC1 solution (2 mg/ml, dissolved in sterile deionized water) was incubated at different temperatures (4, 20, 37, 50, 70, and 90°C) for 1 h, and then the MICs of samples against *E. coli* ATCC25922 were determined.

After the determination of thermal tolerance, thermal stability of cathelicidin-RC1 was examined. Cathelicidin-RC1 solution (2 mg/ml, dissolved in sterile deionized water) was incubated at 37°C for 0 to 96 h. At each time intervals, the MICs of cathelicidin-RC1 against *E. coli* ATCC25922 were determined.

### Cytotoxic and hemolytic assay

The MTT [3-(4-5-dimethylthiazol-2-yl)-2,5-diphenyltetrazolium bromide; Sigma] method was used to determine the *in vitro* cytotoxicity of cathelicidin-RC1 toward two mammalian tumor cell lines (human liver hepatocellular carcinoma cell line HepG2, human prostate cancer cell line PC3) and one normal mammalian cell line (mice fibroblast cell line L929). Cells were cultured in Dulbecco's Modified Eagle's Medium (DMEM, Gibco, USA) supplemented with 10% fetal bovine serum in a humidified 5% CO_2_ atmosphere at 37°C. Cells (approximately 2×10^4^ per well in 100 μl medium) were seeded in 96-well plates and cultured overnight until adhered to the plate. Various concentrations of cathelicidin-RC1 dissolved in the corresponding culture medium were added to the wells and the plates were incubated at 37°C for 48 h. At the end of incubation, 20 μl of MTT solution (5 mg/ml) was added to each well, and the cells were incubated for another 4 h at 37°C. The medium was removed, and 150 μl of dimethyl sulfoxide was added to each well to dissolve the precipitated formazan. The absorbance at 490 nm was measured using a UV microplate autoreader. Cell viability was expressed as the percentage of the negative control group, which was regarded as 100%.

Hemolytic assay was conducted as previously reported [8]. Briefly, human fresh erythrocytes were washed with 0.9% saline for three times and re-suspended to an ultimate concentration of 2% (v/v). Serial dilutions of cathelicidin-RC1 were incubated with the erythrocyte solutions at 37°C for 30 min and then the cells were centrifuged at 2000 rpm for 5 min. The supernatant was removed and the absorbance at 540 nm was measured. 1% Triton X-100 (v/v) was used to determine the 100% hemolysis and 0.9% saline was used as negative control.

### Scanning electron microscopy (SEM)

SEM experiment was used to observe the surface morphology of cathelicidin-RC1-treated bacteria, which can partly reveal the antimicrobial mechanism. The experiment was carried out as described previously [24]. *S. aureus* ATCC25923 and *E. coli* ATCC25922 were cultured in MH broth to exponential phase. After washing with 0.15 M sodium chloride solution for three times, the bacteria were resuspended and incubated with cathelicidin-RC1 (1×MIC) at 37°C for 30 min. The bacteria were centrifuged at 1000 rpm for 10 min, and the pellets were fixed with 2.5% glutaraldehyde solution at 4°C for 2 h. The bacteria were then postfixed in 1% osmium tetroxide for 2 h, dehydrated in a graded series of ethanol, frozen in liquid nitrogen cooled terbutyl alcohol and vacuum dried overnight. After being mounted onto aluminium stubs and vacuum sputter-coated with gold, the samples were observed with a Hitachi S-4800 SEM under standard operating conditions.

### Statistical analysis

The results of MIC values, bacterial killing kinetic assay, salt tolerance, thermal tolerance and thermal stability assay, cytotoxic and hemolytic assay represent the geometric means of three independent experiments.

## Results

### Identification and characterization of bullfrog cathelicidins

**Figure 1:**
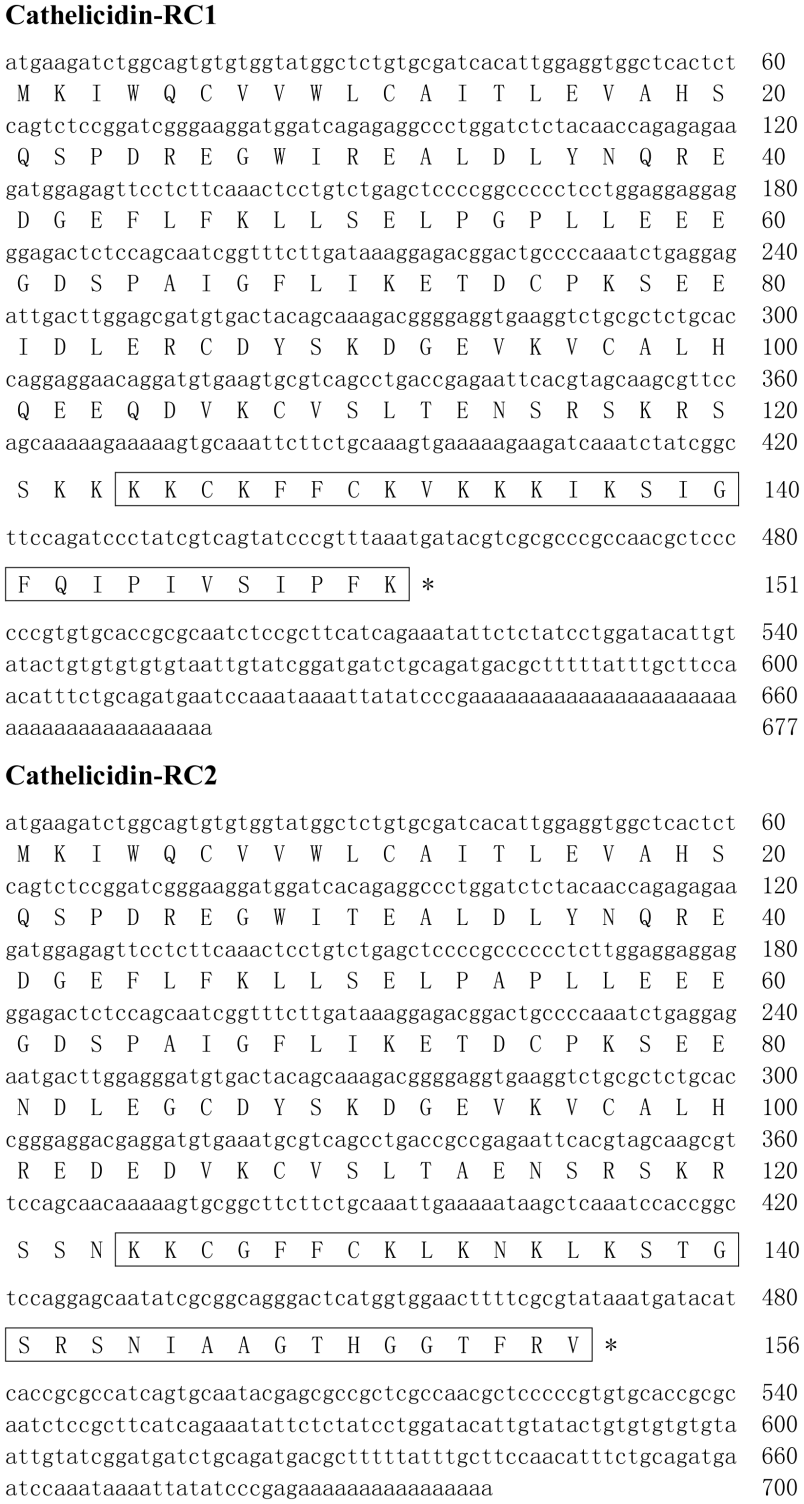
The cDNA sequences encoding cathelicidin-RCs and the predicted prepropeptide sequences. The putative mature peptides of cathelicidin-RCs are boxed. The stop codon is indicated by an asterisk (*).

**Figure 2:**
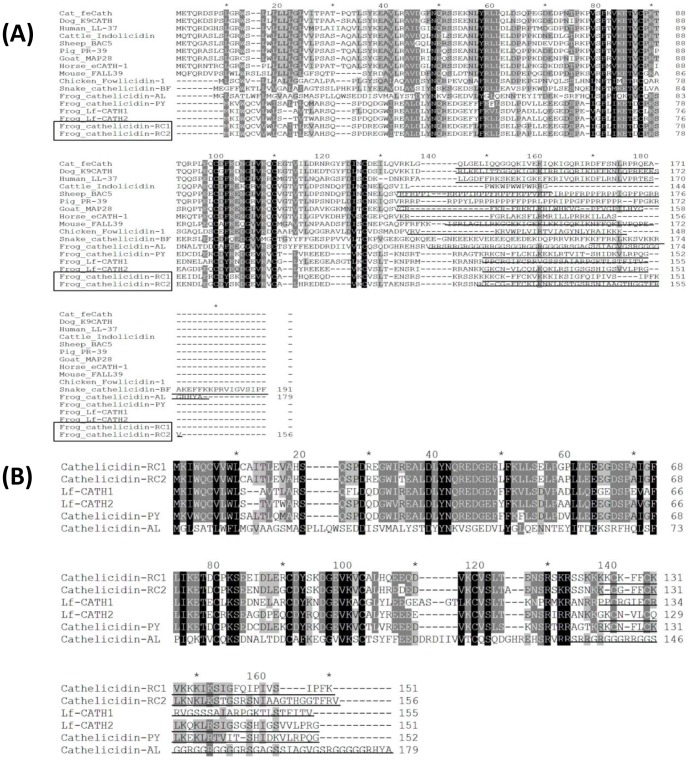
Sequence alignment of cathelicidins. (A) Multi-sequence alignment of cathelicidin-RCs with other representative cathelicidins. (B) Alignment of the amphibian cathelicidins identified so far from *A. loloensisi*, *P. yunnanensis*, *L. fragilis* and *R. catesbeiana*. The identical residues are indicated in black. The highly conserved residues are shaded. Mature peptides of cathelicidins are underlined.

Total RNA was extracted from the lung of *R. catesbeiana* and cDNA library was constructed by a cDNA library construction kit. By PCR-based cDNA cloning method, two cDNAs encoding two different cathelicidins were obtained from the cDNA library (GenBank accession numbers: KF766530-KF766531). The complete nucleotide sequences and translated amino acid sequences of the two cathelicidin precursors were shown in Figure 1. Accordingly, the cDNAs encoding cathelicidin-RC1 and cathelicidin-RC2 precursors are composed of 677 bp and 700 bp, respectively. The translated protein precursors comprise 151 and 156 amino acid residues, respectively. Consistent with other cathelicidins, precursors of cathelicidin-RC1 and cathelicidin-RC2 possess a typical signal peptide sequence, a highly conserved cathelin domain and a cationic C-terminal mature peptide sequence. Multi-sequence alignment of cathelicidin precursors (Fig. 2) indicates that the signal peptide sequences and cathelin domains of cathelicidin-RCs share relatively low similarity with other cathelicidins. However, the four cysteine residues at the end of cathelin domain are highly conserved. Furthermore, amphibian cathelicidins alignment (Fig. 2) indicates that cathelicidin-RCs possess high degree of similarity throughout the entire sequence with previously characterized cathelicidins from frogs, except for cathelicidin-AL from *A. loloensisi* [12].

**Figure 3:**
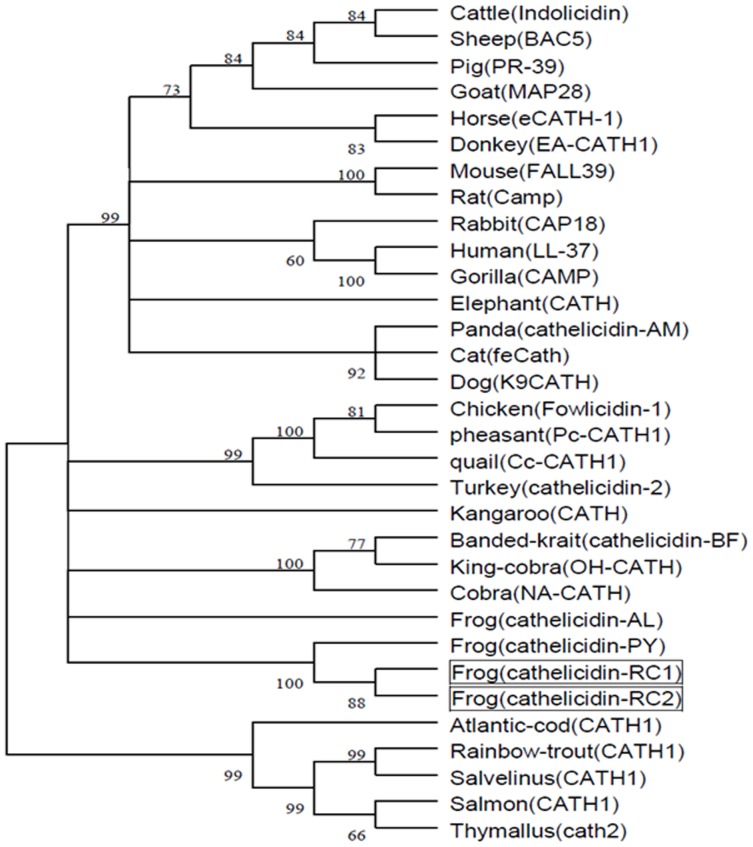
Phylogenetic analysis of vertebrate cathelicidins. The tree was constructed by the neighbor-joining method based on the proportion difference of aligned amino acid sites of the signal peptide and cathelin domain of the prepro-cathelicidin sequences. A total of 1000 bootstrap replicates were used to test the reliability of each branch. The numbers on the branches indicate the percentage of 1000 bootstrap samples supporting the branch. Only branches supported by a bootstrap value of at least 50% are shown. Cathelicidin-RCs are boxed.

Consistent with the previous study [13], the constructed phylogenetic tree in this paper divided cathelicidins into three major clusters, cathelicidins from mammals, cathelicidins from fishes and cathelicidins from birds, reptiles and amphibians (Fig. 3). Cathelicidin-RCs and the other two amphibian cathelicidins used in phylogenetic tree construction (cathelicidin-AL and cathelicidin-PY) were grouped together. Amphibian cathelicidins represent a typical evolutionary transition group between fishes and mammals, and they showed close relationship with the three cathelicidins from snakes (Banded-krait, King-cobra and Cobra).

In the previous paper, Wei et al purified a native amphibian cathelicidin, cathelicidin-PY from the skin secretions of *P. yunnanensis* and successfully cloned its encoded cDNA sequence from the skin cDNA library [13]. According to their study, the mature peptides of cathelicidin-RC1 and cathelicidin-RC2 in the present study were predicted (Fig. 1). Cathelicidin-RC1 is composed of 28 amino acid residues, including 9 basic residues (9 Lysines), and the amino acid sequence is KKCKFFCKVKKKIKSIGFQIPIVSIPFK. Cathelicidin-RC2 is composed of 33 amino acid residues, including 8 basic residues (6 Lysines and 2 Arginines), and the amino acid sequence is KKCGFFCKLKNKLKSTGSRSNIAAGTHGGTFRV. There are two cysteines in the sequences of cathelicidin-RC1 and cathelicidin-RC2. Consistent with cathelicidin-PY, they could form an intramolecular disulfide bridge. The physical and chemical parameters of cathelicidin-RC1 and cathelicidin-RC2 are shown in Table 1.

**Table 1:** Physical and chemical parameters of cathelicidin-RC1 and cathelicidin-RC2.

Peptide	GRAVY	Number of amino acids	Net charge	Theoretical pI	Mw
Cathelicidin-RC1	0.118	28	9+	10.32	3282.2
Cathelicidin-RC2	−0.479	33	8+	10.67	3541.2

GRAVY: grand average of hydropathicity.

### Secondary structure modeling and determination of cathelicidin-RCs

**Figure 4:**
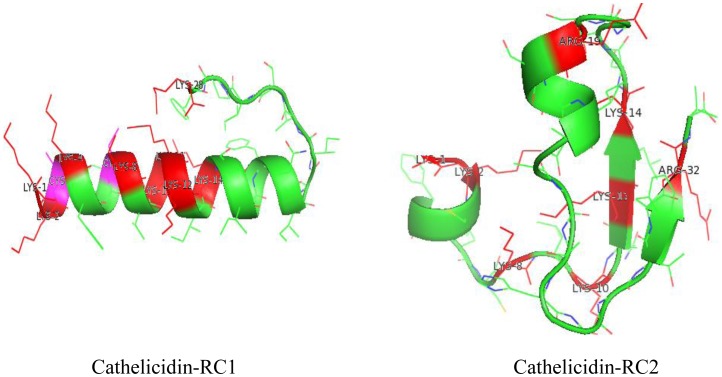
Secondary structure modeling of cathelicidin-RCs. The models of cathelicidin-RCs were produced by Mod6v2 version of MODELLER. Visualization of the structures were accomplished by Pymol and represented in the form of ribbons. The homology modeled structures were displayed in green. Residues of Lysines and Arginines were labeled in red and Cysteines were labeled in purple in shortened forms.

Homology structure modeling of cathelicidin-RCs was produced by MODELLER (version Mod6v2). Visualization of the structures were accomplished by Pymol and represented in the form of ribbons. BLAST search against PDB protein databank indicated that *E. coli* ribonucleotide reductase (PDB entry 2AV8) and human sorbitol dehydrogenase (PDB entry 1PL6) possessed the maximum sequence similarity with cathelicidin-RC1 and cathelicidin-RC2, respectively. As a result, they were selected as templates for structure modeling analysis. As shown in Fig. 4, the homology modeled structures were displayed in green. Residues of Lysines and Arginines were labeled in red in shortened forms. Cysteines were also labeled in purple but the intramolecular disulfide bridge was not marked out. The N-terminal region of cathelicidin-RC1 exhibits amphipathic alpha-helical conformation, which implies that cathelicidin-RC1 is one member of membrane-active antimicrobial peptides. The homology modeled structure of cathelicidin-RC2 exhibited a helix-strand-helix-strand conformation. The two parallel strands on the right were flanked by the two alpha-helices on the left side. The 8 basic residues evenly distributed in the entire molecule but no structural amphipathicity presented, which implied the low antimicrobial activity of cathelicidin-RC2. AMPs, regardless of which groups, generally assume an amphipathic conformation, in which clusters of hydrophobic and cationic amino acids are spatially organized in discrete sectors of the molecules [1]. The cationic charge and amphipathicity facilitates AMPs adhering to the negatively charged microbial membranes and interacting with the fatty acyl chains [5].

**Figure 5:**
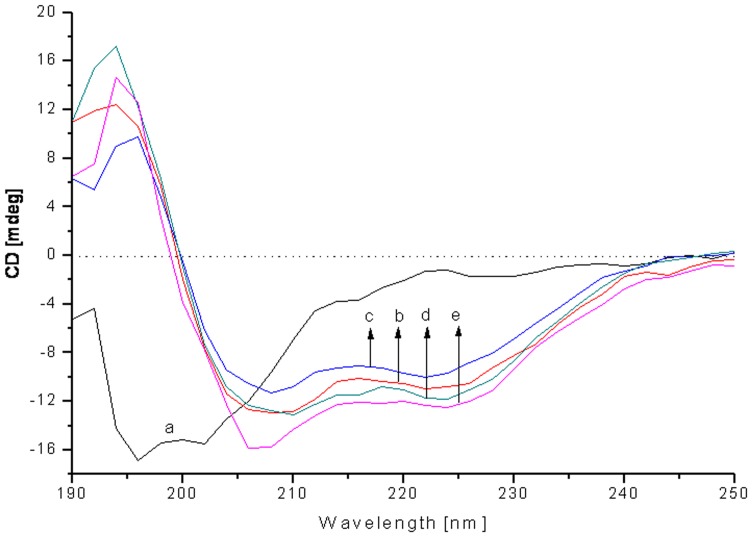
Circular dichroism analysis of cathelicidin-RC1 in different solvent environments. a∼e: in SDS micelles of 0, 30, 60, 90, 120 mM.

The secondary structure of cathelicidin-RC1 in different solvent environments was further confirmed by CD spectroscopy (Fig. 5). The CD spectra of cathelicidin-RC1 dissolved in H_2_O showed a strong negative peak at 200 nm, indicating that cathelicidin-RC1 adopted a random-coil conformation in H_2_O. In the membrane-mimetic environment of SDS/H_2_O solutions (30–120 mM), the CD spectra showed two negative peaks at 208 nm and 222 nm, which indicated that the main secondary structure component of cathelicidin-RC1 dissolved in membrane-mimetic environment was alpha-helix. The secondary structure constitution of cathelicidin-RC1 dissolved in 60 mM SDS/H_2_O solution was calculated as 44.8% alpha-helix, 12.2% beta-sheet, 14.9% beta-turn and 26.3% random-coil.

### Antimicrobial activity of cathelicidin-RCs

In order to test the antimicrobial activity, cathelicidin-RC1 and cathelicidin-RC2 were chemically synthesized and the purity was confirmed to be >98%. The MICs of the two peptides against 48 microorganisms, including Gram-positive bacteria, Gram-negative bacteria and fungi were determined. As listed in Table 2, cathelicidin-RC1 exhibited potent and broad-spectrum antimicrobial activity (MICs ranging from 1.43 to 22.85 μM) against most of the tested microorganisms. As expected, the positive control meropenem, a new generation of carbapenem antibiotic, was more effective than cathelicidin-RC1, with MICs ranging from 0.03 to 48.9 μM. However, the traditional antibiotic ampicillin was less effective than cathelicidin-RC1 and meropenem (MICs ranging in 12.62–201.9 μM), especially 28 of the 48 tested microorganisms showed resistance to ampicillin. Unlike cathelicidin-RC1, cathelicidin-RC2 displayed very weak antimicrobial activity. It was only active against two Gram-negative bacteria, *Proteus mirabilis* clinical strain and *S. maltophilia* clinial strain 2, with MICs up to 42.36 μM.

**Table 2:** Antimicrobial activity of cathelicidin-RC1 and cathelicidin-RC2.

Microorganisms	MIC (μg/ml)			
	Cathelicidin-RC1	Cathelicidin-RC2	Meropenem	Ampicillin
Gram-negative bacteria				
*Escherichia coli* ATCC25922	4.69 (2.86 μM)	>200	0.01 (0.03 μM)	4.69 (12.62 μM)
*E. coli* clinical strain 1	9.38 (2.86 μM)	>200	0.12 (0.31 μM)	9.38 (25.24 μM)
*E. coli* clinical strain 2	18.75 (5.71 μM)	>200	0.06 (0.15 μM)	>200
*E. coli* clinical strain 3	18.75 (5.71 μM)	>200	0.03 (0.08 μM)	>200
*E. coli* clinical strain 4	18.75 (5.71 μM)	>200	0.12 (0.31 μM)	>200
*Shigella dysenteriae* clinical strain	4.69 (1.43 μM)	>200	0.03 (0.08 μM)	75 (201.9 μM)
*Klebsiella peneumoniae* clinical strain 1	37.5 (11.43 μM)	>200	0.06 (0.15 μM)	>200
*K. peneumoniae* clinical strain 2	18.75 (5.71 μM)	>200	0.06 (0.15 μM)	>200
*K. peneumoniae* clinical strain 3	18.75 (5.71 μM)	>200	0.06 (0.15 μM)	>200
*K. peneumoniae* clinical strain 4	18.75 (5.71 μM)	>200	0.06 (0.15 μM)	>200
*K. peneumoniae* clinical strain 5	>200	>200	0.12 (0.31 μM)	>200
*K. peneumoniae* clinical strain 6	37.5 (11.43 μM)	>200	0.12 (0.31 μM)	>200
*K. peneumoniae* clinical strain 7	37.5 (11.43 μM)	>200	0.12 (0.31 μM)	>200
*K. peneumoniae* clinical strain 8	75 (22.85 μM)	>200	0.12 (0.31 μM)	>200
*Serratia marcescens* clinical strain	>200	>200	0.06 (0.15 μM)	37.5 (100.9 μM)
*Klebsiella oxytoca* clinical strain	4.69 (1.43 μM)	>200	0.12 (0.31 μM)	>200
*Proteus vulgaris* clinical strain	>200	>200	0.23 (0.61 μM)	18.75 (50.49 μM)
*Proteus mirabilis* clinical strain	18.75 (5.71 μM)	150 (42.36 μM)	0.47 (1.22 μM)	75 (201.9 μM)
*Acinetobacter baumannii* clinical strain 1	>200	>200	0.23 (0.61 μM)	37.5 (100.9 μM)
*A. baumannii* clinical strain 2	>200	>200	0.23 (0.61 μM)	9.38 (25.24 μM)
*Stenotrophomonas maltophilia* clinical strain 1	37.5 (11.43 μM)	>200	4.69 (12.22 μM)	18.75 (50.49 μM)
*S. maltophilia* clinial strain 2	9.38 (2.86 μM)	150 (42.36 μM)	4.69 (12.22 μM)	>200
*Pseudomonas aeruginosa* ATCC27853	9.38 (2.86 μM)	>200	0.06 (0.15 μM)	>200
*P. aeruginosa* clinical strain 1	18.75 (5.71 μM)	>200	0.06 (0.15 μM)	9.38 (25.24 μM)
*P. aeruginosa* clinical strain 2	4.69 (1.43 μM)	>200	1.88 (4.89 μM)	>200
*Salmonella paratyphi A* clinical strain	37.5 (11.43 μM)	>200	0.12 (0.31 μM)	>200
Gram-positive bacteria				
*Staphylococcus aureus* ATCC25923	>200	>200	0.06 (0.15 μM)	9.38 (25.24 μM)
*S. aureus* clinical strain 1	9.38 (2.86 μM)	>200	0.06 (0.15 μM)	18.75 (50.49 μM)
*S. aureus* clinical strain 2	9.38 (2.86 μM)	>200	0.12 (0.31 μM)	>200
*S. aureus* clinical strain 3	>200	>200	18.75 (48.9 μM)	>200
*S. aureus* clinical strain 4	>200	>200	0.12 (0.31 μM)	9.38 (25.24 μM)
*S. aureus* clinical strain 5	4.69 (1.43 μM)	>200	0.06 (0.15 μM)	9.38 (25.24 μM)
*Bacillus cereus* clinical strain	>200	>200	0.06 (0.15 μM)	>200
*Bacillus subtilis* clinical strain	9.38 (2.86 μM)	>200	0.03 (0.08 μM)	75 (201.9 μM)
*Enterococcus faecium* clinical strain	9.38 (2.86 μM)	>200	9.38 (24.45 μM)	>200
*Nocardia asteroides* clinical strain	75 (22.85 μM)	>200	4.69 (12.22 μM)	18.75 (50.49 μM)
*Enterococcus faecalis* clinical strain	75 (22.85 μM)	>200	9.38 (24.45 μM)	37.5 (100.9 μM)
*Staphylococcus epidermidis* clinical strain	9.38 (2.86 μM)	>200	0.94 (2.45 μM)	>200
Fungi				
*Candida albicans* clinical strain 1	9.38 (2.86 μM)	>200	0.12 (0.31 μM)	>200
*C. albicans* clinical strain 2	>200	>200	0.23 (0.61 μM)	>200
*C. albicans* clinical strain 3	37.5 (11.43 μM)	>200	0.06 (0.15 μM)	>200
*C. albicans* clinical strain 4	9.38 (2.86 μM)	>200	0.12 (0.31 μM)	18.75 (50.49 μM)
*C. albicans* clinical strain 5	>200	>200	0.06 (0.15 μM)	>200
*C. albicans* clinical strain 6	>200	>200	0.03 (0.08 μM)	>200
*Candida glabrata* clinical strain 1	9.38 (2.86 μM)	>200	0.03 (0.08 μM)	18.75 (50.49 μM)
*C. glabrata* clinical strain 2	>200	>200	0.06 (0.15 μM)	>200
*Cryptococcus neoformans* clinical strain	>200	>200	0.47 (1.22 μM)	4.69 (12.62 μM)
*Arcyria cinerea*	18.75 (5.71 μM)	>200	0.12 (0.31 μM)	>200

### Bacterial killing kinetics of cathelicidin-RC1

Using meropenem as positive control, killing kinetics of cathelicidin-RC1 against *E. coli* ATCC25922 was investigated by a colony counting method. As illustrated in Table 3, at a concentration of 5×MIC, cathelicidin-RC1 rapidly exerted its antimicrobial function. It just took less than 45 min for cathelicidin-RC1 to kill all the *E. coli* ATCC25922 cells. More importantly, the colony forming units (CFUs) remained zero when the incubation time extended to 180 min, which implied that the antimicrobial property of cathelicidin-RC1 was lethal. In contrast, at the same concentration of 5×MIC, it took at least 120 min for the positive control meropenem to completely kill the *E. coli* ATCC25922 cells.

**Table 3:** Killing kinetics of cathelicidin-RC1 against *E. coli* ATCC25922.

Time	Colony Forming Units (×10^3^, CFUs/ml)								
	0 min	10 min	20 min	30 min	45 min	60 min	90 min	120 min	180 min
Cathelicidin-RC1	83±7.9	79±21.2	40±28.1	50±10.1	8±5.6	0±0.0	0±0.0	0±0.0	0±0.0
Meropenem	78±8.7	98±11.7	82±12.1	90±14.9	63±40.7	69±15.5	1±1.7	0.3±0.6	0±0.0
Control	93±8.1	81±20.8	96±10.1	104±30.5	96±42.6	168±17.9	387±20.1	637±115.8	1104±145.7

*E. coli* ATCC25922 was mixed with samples at concentration of 5×MIC for 0, 10, 20, 30, 45, 60, 90, 120 and 160 mins. The results represent mean values of three independent experiments performed in duplicates. The MICs of cathelicidin-RC1 and meropenem against *E. coli* ATCC25922 are 4.69 and 0.01 μg/ml, respectively.

### Salt tolerance, thermal tolerance and thermal stability of cathelicidin-RC1

Previous studies have indicated that the antimicrobial activities of many AMPs differ greatly in the presence or absence of salt [25]–[27]. Therefore, influence of salt on the antimicrobial efficacy of cathelicidin-RC1 was examined. As shown in Table 4, in the presence of 50 and 100 mM sodium chloride, the MICs remained at 4.69 μg/ml. At the human physiological salt concentration (150 mM sodium chloride), cathelicidin-RC1 maintained its relatively high killing activity, since the MIC value was increased only by a factor of two (9.38 μg/ml) compared with the no-salt condition. Moreover, at a salt concentration up to 400 mM, the MIC was merely fourfold as high (18.75 μg/ml) compared with the no-salt condition. The strong salt tolerant property of cathelicidin-RC1 implies its potential for systemic therapeutic applications.

**Table 4:** Salt tolerance of the antimicrobial activitiy of cathelicidin-RC1.

NaCl concentration (mM)	MIC (μg/ml)
0	4.69
50	4.69
100	4.69
150	9.38
200	9.38
400	18.75

The salt tolerance of cathelicidin-RC1 was tested against *E. coli* ATCC25922 by measuring the MICs in the presence of 0, 50, 100, 150, 200 and 400 mM sodium chloride. The results represent mean values of three independent experiments performed in duplicates.

As an important class of natural bio-active molecules, AMPs are easily degraded by physical and biological factors. Thermal tolerance is an essential characteristic for AMPs to be carefully investigated in application. As listed in Table 5, cathelicidin-RC1 retained its potent activity after incubated in a series of temperatures (4-90°C) for 1 h, although the MICs slightly increased from 4.69 to 9.38 μg/ml.

**Table 5:** Thermal tolerance of cathelicidin-RC1.

Temperature (°C)	MIC (μg/ml)
4	4.69
20	4.69
37	4.69
50	9.38
70	9.38
90	9.38

Thermal tolerance of cathelicidin-RC1 was determined by measuring MICs after incubation at different temperatures. Cathelicidin-RC1 solution (2 mg/ml, dissolved in sterile deionized water) was incubated at different temperatures (4, 20, 37, 50, 70, and 90°C) for 1 h, then the MICs of the peptides against *E. coli* ATCC25922 were determined. The results represent mean values of three independent experiments performed in duplicates.

Some traditional antibiotics, such as cephalosporins, are extremely unstable in solutions, which significantly restrict their usage. In contrast, cathelicidin-RC1 solution exhibited high stability (Table 6). After placed at 37°C for 96 h, the MIC value of cathelicidin-RC1 against *E. coli* ATCC25922 remained at 9.38 μg/ml.

**Table 6:** Thermal stability of cathelicidin-RC1 solution at 37°C.

Time (h)	MIC (μg/ml)
0	4.69
6	4.69
12	4.69
24	4.69
48	4.69
72	9.38
96	9.38

Cathelicidin-RC1 was dissolved in sterile deionized water to an ultimate concentration of 2 mg/ml and stored at 37°C for 0 to 96 h. At different time intervals, cathelicidin-RC1 samples were taken and the antimicrobial activities of samples against *E. coli* ATCC25922 were determined using a two-fold microdilution method. The results represent mean values of three independent experiments performed in duplicates.

### Cytotoxicity and hemolysis of cathelicidin-RC1

Two mammalian tumor cell lines (human liver hepatocellular carcinoma cell line HepG2, human prostate cancer cell line PC3) and one mammalian normal cell line (mice fibroblast cell line L929) were used to evaluate the cytotoxicity of cathelicidin-RC1. Cathelicidin-RC1 showed slight cytotoxic effect towards the three tested cell lines. At a concentration up to 200 μg/ml, cathelicidin-RC1 merely induced 4.3, 2.5, and 1.1% death of HepG2, PC3 and L929 cells, respectively.

To evaluate the hemolytic activity of cathelicidin-RC1, human fresh erythrocytes were used in the experiment. No apparent hemolytic activity was observed for cathelicidin-RC1. At concentrations of 100 and 200 μg/ml, cathelicidin-RC1 induced 0.2 and 2.6% human erythrocyte hemolyisis, respectively.

### Effect of cathelicidin-RC1 on microbial membrane morphology

**Figure 6:**
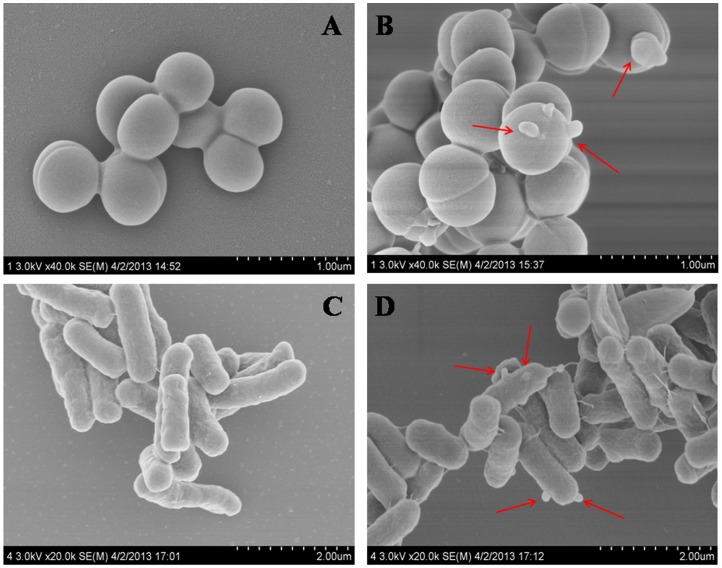
Scanning electron microscopy analysis of cathelicidin-RC1-treated bacteria. (A) Control *S. aureus* ATCC25923; (B) Cathelicidin-RC1-treated *S. aureus* ATCC25923; (C) Control *E. coli* ATCC25922; (D) Cathelicidin-RC1-treated *E. coli* ATCC25922. The arrows indicate damage to the microbial membranes of bacteria or the intracellular inclusions efflux.

Previous studies have demonstrated that the microbial membrane is a key target for many cathelicidins [28], [29]. Disruption of the microbial membrane integrity results in obvious membrane morphological alteration, which can be clearly observed by SEM. As illustrated in Fig. 6, the untreated *S. aureus* and *E. coli* cells possessed normal shape and smooth surfaces (Fig. 6A, C). After treated with cathelicidin-RC1 (1×MIC) for 30 min, the cell surface appeared obvious morphological alterations (Fig. 6B, D). The membrane integrity of cells seems to be disrupted and a large number of filaments appeared on the surface of *E. coli* cells.

## Discussion

The widespread emergence of drug resistance in microbial pathogens, which is ascribed to the extensive abuse of antibiotics, seriously threatens public health. As a result, there is an urgent need for the development of novel antimicrobial agents. Since the initial discovery in 1980s, AMPs have been regarded as promising alternatives to conventional antibiotics [30]. To date, dozens of AMPs or analogs have been applied to commercial development [31]. Among them, three cathelicidin analogs are undergoing clinical trials. Omiganan (MBI-226, CPI226), an analogue of the cattle indolicidin, is under Phase 3 clinical study for topical skin antisepsis in healthy adult subjects and treatment of local catheter site infection in patients with central venous catheters [32]. MX-594AN, another analogue of indolicidin, is undergoing Phase 2 trial for the treatment of papulopustular rosacea [31]. Iseganan (IB-367), an analogue of the pig protegrin-1, is undergoing Phase 3 clinical trial in preventing oral mucositis in patients who are receiving radiation therapy for head and neck cancer [33]. Small size, good stablity, broad-spectrum activity, special mechanism and slight possibility to induce resitance, these features make cathelicidins good candidates for the development of novel peptide antibiotics. As a result, exploring novel cathelicidins from all sorts of vertebrates is meaningful for peptide antibiotic design.

In the present study, two novel cathelicidins (cathelicidin-RC1 and cathelicidin-RC2) were identified from the lung of bullfrog *R. catesbeiana*. The cloned cDNAs encoding cathelicidin-RC1 and cathelicidin-RC2 are 677 and 700 bp in length, respectively (Fig. 1). And the translated precursors contain 151 and 156 amino acid residues, respectively. The precursors of cathelicidin-RCs possess the conserved structural organization of cathelicidins, including a signal peptide, a highly conserved cathelin domain and a C-terminal mature peptide. Moreover, the precursors of cathelicidin-RCs retain the four conserved cysteines embedded within the C-terminus of cathelin domain, which is a typical characteristic of cathelicidin family AMPs [Fig. 2(A)]. According to the inherent numbers of cathelicidin genes, vertebrate species can be divided into two groups: “polycathelicidin species”, which contain different gene-clusters encoding several different cathelicidins, and “monocathelicidin species”, which possess a single cathelicidin gene [34]. Previous studies about amphibian cathelicidins indicated that ranid frogs may be “monocathelicidin species” [12], [13]. However, our previous study about Lf-CATHs from *L. fragilis* [14] and the identification of cathelicidin-RCs in the present study verify that ranid frogs should be “polycathelicidin species”. The screening of cDNAs encoding frog cathelicidins in the previous studies may be unsufficient. Using the signal peptide and cathelin domain sequences of representative cathelicidins deposited in NCBI database, a phylogenetic tree was constructed [Fig. 3]. Accordingly, cathelicidin-RCs and cathelicidin-AL and cathelicidin-PY were grouped together in a subordinate branch. From the bottom to the top of the phylogenetic tree, the branches successively represent fishes, amphibians, reptiles, birds and mammals, which are consistent with the course of animal evolution. This implies that cathelicidin genes may be ideal candidates for the molecular evolution study of vertebrates.

The result of sequence alignment of the amphibian cathelicidins indicated that the deduced mature cathelicidin-RCs possess similar primary structural features as other amphibian cathelicidins (except for cathelicidin-AL), especially the two conserved cysteines at the N-terminus [Fig. 2(B)]. The cysteines form an intramolecular disulfide bridge (Cys3-Cys7) at the N-terminus, and the disulfide loop is only composed of five residues. Most of the other residues are extended in the C-terminal of the disulfide loop [13]. Secondary structure prediction of cathelicidin-RCs was also performed. As predicted, cathelicidin-RC1 mainly assumes an amphipathic alpha-helical conformation, which is the most common spatial arrangement among cathelicidin family AMPs (Fig. 4) [35]. In order to verify the validity of the prediction, CD experiment was carried out. The results indicated that in H_2_O, cathelicidin-RC1 adopts a random-coil conformation, but in the membrane-mimetic environments of SDS/H_2_O solutions, cathelicidin-RC1 changes its conformation into alpha-helix, which facilitates its insertion into lipid membrane (Fig. 5). On the contrary, homology modeling analysis predicted that cathelicidin-RC2 adopts a helix-strand-helix-strand conformation and it could not form a stable amphipathic conformation, which could partly explain why cathelicidin-RC2 does not possess potent antimicrobial activity (Fig. 4).

The data of antimicrobial assay indicated that cathelicidin-RC1 possesses potent and broad-spectrum antimicrobial activity against the tested microorganisms (Table 2). Among the 48 microorganism strains, 34 strains could be killed by cathelicidin-RC1 with MICs ranging from 4.69 to 75 μg/ml. For several strains, cathelicidin-RC1 even exhibited better efficacy than the positive control meropenem. To our knowledge, cathelicidin-RC1 represents one of the most potent cathelicidins discovered to date. In contrast, cathelicidin-RC2 didn't exhibit effective activity against the 48 tested microorganisms. It was only active against two of them (*P. mirabilis* clinical strain and *S. maltophilia* clinical strain 2), with MICs up to 150 μg/ml. Numerous studies have indicated that cathelicidins are multifunctional peptides of the innate immunity [19]. Besides direct antimicrobial activity, some cathelicidins also possess other biological functions, such as neutrophils chemotaxis [36], [37], transcriptional responses alteration [38], promotion of wound repair [39], and induction of angiogenesis [40]. Therefore, a plausible scenario would be that cathelicidin-RC1 and cathelicidin-RC2 play different roles in the host defense immunity of bullfrog. Cathelicidin-RC1 is mainly responsible for the direct clearance of invaded pathogens, while cathelicidin-RC2 participates in some unknown immune response.

Owing to the potent antimicrobial activity, cathelicidin-RC1 was selected for further structural and functional analysis. Cathelicidin-RC1 exhibited rapid bacteria-killing efficacy. It could kill the tested *E. coli* within 45 min, which is far more rapid than meropenem (at least 120 min) (Table 3). Traditional antibiotics generally are stable when lyophilized into powder. But after being dissolved in solvents, some of them become unstable and quickly lose their activities in a short time, which significantly restrict their usage. Comparatively, the antimicrobial activity of cathelicidin-RC1 was extremely stable. After being incubated at 90°C for 1 h or at 37°C for 96 h, cathelicidin-RC1 solution still retained its potent activity (Table 5, 6). Besides, unlike some of the other AMPs, the antimicrobial activity of cathelicidin-RC1 was highly salt-tolerant, it maintained its potent killing activity even beyond physiological salt concentrations (Table 4). Furthermore, cathelicidin-RC1 was not cytotoxic towards mammalian normal and tumor cells, which are important advantages for its application.

Similar as other cathelicidins, the positively charged and amphipathic alpha-helical structure of cathelicidin-RC1 facilitates its binding and permeating into the negatively charged bacterial phospholipid membranes, which finally results in rapid rupture of microbial cells. The results of SEM analysis suggest the probable rupture of bacterial cells. Therefore, we deduce that the antimicrobial action of cathelicidin-RC1 is attributable to the disruption of microbial membrane.

In summary, in the present study, we report the identification and characterization of two novel cathelicidin family AMPs, cathelicidin-RC1 and cathelicidin-RC2, from the bullfrog *R. catesbeiana*. Both cathelicidin-RC1 and cathelicidin-RC2 exhibited low sequence similarity with the known cathelicidins. Unlike most of the other cathelicidins, cathelicidin-RC2 showed weak antimicrobial activity against the tested microbial strains. In contrast, cathelicidin-RC1 exhibited potent, broad-spectrum, rapid, salt-independent and highly stable antimicrobial activity. Besides, it also exhibited low cytotoxic activity toward mammalian cells. All of these properties make cathelicidin-RC1 an ideal template for the development of novel peptide antibiotics.
